# Uppsala Clinical Research Center—development of a platform to promote national and international clinical science

**DOI:** 10.1080/03009734.2018.1540506

**Published:** 2018-12-04

**Authors:** Lars Wallentin, Bertil Lindahl

**Affiliations:** Uppsala Clinical Research Center, Uppsala University, Uppsala, Sweden

**Keywords:** Biobank, biomarkers, clinical research, clinical trials, genetics, health economy, quality development, registries

## Abstract

Uppsala Clinical Research Center (UCR) is a non-profit organization that provides service for clinical research aiming for development and improvement of health care in Sweden and worldwide. UCR was started in 2001 with the ambition to shift the focus of clinical research from new medications or devices launched by the industry to problem-based research on issues identified in clinical reality, for example through the national quality registries. In order to accomplish these goals, UCR has established services in: 1) clinical trials of new and old methods in health care; 2) quality development of the health care system supported by internet-based national quality registries; 3) biostatistics, epidemiology, and data management; 4) biobanking of biological materials (Uppsala Biobank); 5) high-throughput biochemical analyses (UCR laboratory); and 6) academic leadership by the members of the UCR research faculty. The UCR clinical trials group provides services for investigator-driven projects in all areas of health care, for global mega-trials on new pharmaceutical treatments and devices, for biobanking including biomarker and genetics analyses, and for clinical events adjudication in national as well as global mega-trials. During the last few years, UCR has been a pioneer in establishing the registry-based randomized clinical trial (R-RCT), which today is an international model on how to perform cost-effective pragmatic randomized trials in the real-world environment. In 2002, UCR started the first national competence center for national quality registries, which pioneered the development of the current internet-based technologies for registering, reporting, and supporting continuous systematic improvement of health care. UCR is currently harboring around 20 national quality registries in all areas of health care. Today, UCR is the leading European center for registry-based quality development and evaluation of new medical treatments in cardiovascular care and has started to support other European countries in implementing the UCR registry platform in order to improve quality of care in the European Union.

## Introduction

Uppsala Clinical Research Center (UCR) is a non-profit organization that supports research and quality development in health care in Sweden and internationally. The center was founded on 1 July 2001, based on a proposal from the authors of this manuscript. The reasons for the creation of UCR were the needs to support the nationally and internationally successful clinical research groups on cardiovascular, metabolic, cancer, and infectious diseases, and the close collaboration of these research groups with the advanced clinical and basic science laboratories in Uppsala (1–4). There was also an urgent need to support the national quality registries in cardiovascular care, which at this time pioneered the utilization of internet-based technologies as well as quality development and research in this area (5–7). At this time, clinical research in Sweden was in a period of crisis because of low status for medical science and medical faculties, few opportunities for systematic training and education, few career positions, and decreasing grant funding (Den kliniska forskningens kris och pris, MFR-rapport 5; 1998 (ISBN:91-85547-12-3)). Clinical research projects were often limited to either being a minor contributor in industry-related international mega-trials or performing local, underpowered, underfunded research projects with small chances to produce results with international impact.

**Figure 1:**
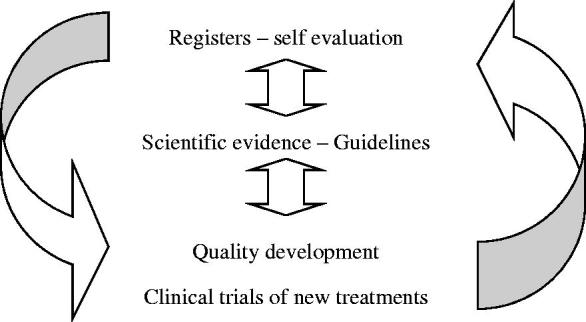
Evidence-based health care development. UCR’s main objective is to provide services for clinical research aiming for development and improvement of health care based on UCR’s combination of systematic monitoring and continuous evaluation of health care performances and outcomes in registers together with performance of prospective randomized clinical trials of new treatments and finally support of implementation by quality development programs; this has led to the new concept ‘evidence-based health care development’.

UCR was started with the ambition to shift the focus of clinical research from new medications or devices launched by the industry to problem-based research on issues identified in clinical reality, for example through the national quality registries. The objectives of clinical research needed to be renewed by updating the knowledge on occurrence and natural courses of disease, utilization of old and new treatments, and their effects in real-life health care. Both old and new methods for diagnosis, prognostication, decision support, and treatment should be continuously evaluated by scientific methods. A similar systematic scrutiny should be applied for interventional treatments with surgery, endoscopic, and catheter-based treatments as for new medications. The ambition of UCR was to support the development of a systematic learning process, which should also facilitate a better controlled introduction of new methods and elimination of old ones in the health care system (Figure 1). In addition, a modern scientific approach was also needed to support the inclusion of issues on quality of life, health economy, and prioritization in all clinical research projects (Table I).

**Table I.** Planned services at the foundation of UCR.

Computer- and internet-based information and communication
Data management for registries and clinical studies
Statistical advice, consultation, and computation services
Epidemiological analyses
Behavioral science service
Project management
Quality control
Biobank service
Biochemical laboratory service
Presentation and publication service
Economic assistance and legal support
Methodological research development
Courses and tutoring in research and clinical trial methodology
Coordination of regional, national, and international research projects
New focus areas of clinical research: Registry-based research Registry-based clinical trials Translational research Implementation research Disease-oriented research Organizational research Care-oriented research Research in primary care Patient-related outcomes

**Figure 2:**
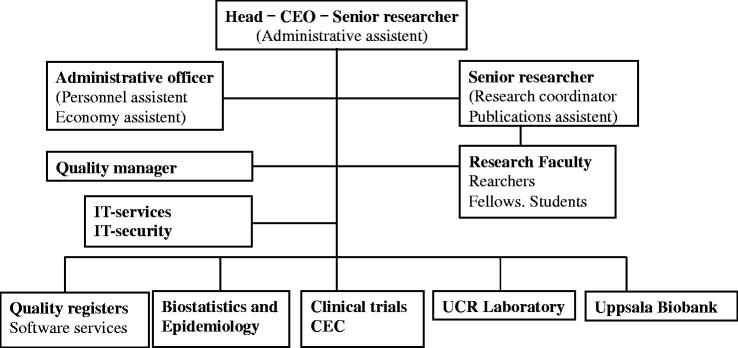
UCR organization 2013.

From the start, UCR has had the same overriding main objective, which is to provide service for clinical research aiming to develop and improve health care (Figure 1). UCR has had its main activities in six areas (Figure 2). The main reason for the development of UCR was the universally recognized need for improved services in (i) clinical trials of new and old methods in health care. However, the first leaders of UCR also took the, at that time rather unique, initiative to include also (ii) quality development of the health care system supported by internet-based national quality registries as an equally important part of the services. As for all other research services (iii) biostatistics, epidemiology, and data management were also important components of the initial organization. Over the years, the UCR organization has also integrated services for (iv) biobanking of biological materials (Uppsala Biobank) and (v) high-throughput biochemical analyses in plasma samples from large international clinical trials and observational materials (UCR laboratory). Since the start of UCR both the whole organization and most individual projects have been initiated and led by members of the (vi) UCR research faculty.

As cardiologists started UCR, the three UCR directors as well as most faculty members have been senior researchers in the cardiovascular area. The faculty has gradually been growing to include researchers at all levels from senior professors to research students whose presence at UCR enriches the discussions and acts both as a solid foundation for ongoing projects and as a spearhead for future innovative activities. The UCR faculty group is also the hub for the extensive networking with other national and international researchers and research centers who are often the key contacts for new projects. Over the years, the activities at UCR have gradually expanded and included researchers also in other areas, for example cancer, neurology and care of the elderly, diabetes, nutrition, psychiatry, infection, radiology, and pulmonary, gastrointestinal, and orthopedic diseases.

### Clinical trials

In order to perform academic clinical trials, it is today almost a necessity to have support from a clinical research center. Very few clinical trialists can keep the competence needed to perform larger clinical trials within their own organization. Without this support, today’s complicated regulation around clinical trials will form an almost insurmountable obstacle even for the startup of any investigator-initiated trial. In order for a clinical trials center to manage planning, performance, analysis, and reporting of larger multi-center clinical trials, there is a need for a critical mass of personnel and technical resources within several different areas. Therefore, the support for clinical trials concerning new or old methods of diagnosis and treatment is a key part of the development of UCR.

The UCR clinical trials group started with two project managers and three research nurses and has over the years grown to a considerably larger group of around 50 persons. Today, the group provides complete services including study design, protocols, patient information, negotiations, agreements, contracts, applications to authorities, recruitment of countries, centers and investigators, case report form (CRF) and internet-based remote data entry, monitoring and quality assurance, planning and management of biological samples including transportation and storing in the UCR biobank, biochemical and genetic analyses, project coordination, logistics, administration and archiving, consultation, and education. In addition, during the first 10 years UCR was running the UCR clinic, which performed and supported clinical trials in healthy volunteers and outpatients.

The primary purpose for the clinical trials group is to support physicians and other investigators, especially in investigator-initiated and registry-based clinical trials. The same services are also provided for clinical trials in collaboration with external sponsors. The group has been working on investigator-initiated projects within many different areas: cardiovascular disease, internal medicine, obstetrics, rheumatology, renal disease, physiology, odontology, nutrition, cancer, ear–nose–throat diseases, infectious diseases, psychology, and quality development. The projects concern research on pharmaceutical agents, functional foods, medical implants and devices, quality development methods, and national quality registries. They have been, and are, performed in collaboration with governmental departments, Swedish Board of Health and Welfare, Swedish Federation of County Councils, Swedish heart–lung foundations, Swedish Cancer Foundation, Center for Clinical Trials of Foods, and pharmaceutical and medical device companies.

The international breakthrough for the clinical trials group was being one of the leading centers in the development and performance of four global phase III mega-trials in the cardiovascular area, each including 15,000 to 19,000 patients: the RELY (8), PLATO (9), ARISTOTLE (10), and STABILITY (11) trials which were started between 2005 and 2007. Through these trials, UCR has become an appreciated member of a global network of academic research organizations, VIGOUR, which is continuously taking on new assignments in international clinical trials and has its center at Duke Clinical Research Institute (DCRI) in Durham, NC, USA (12). A second breakthrough by these trials was the establishment of the UCR laboratory in 2008, which was a necessity as UCR researchers took the initiative and the responsibility for an extensive biomarker and genetic substudy program with analyses of blood samples from all patients in these mega-trials (13–18). In addition, the large repository of samples from these trials substantially contributed to the third breakthrough, the establishment of Uppsala Biobank in 2008, which was later integrated with the UCR organization. A fourth breakthrough by participation in these mega-trials was the need to set up a center for clinical events adjudication (CEC) (19). This CEC organization has thereafter rapidly expanded and become one of the world-leading centers for CEC, with assignments for these services in many international trials. Finally, it should be emphasized that a less recognized fifth breakthrough occurred during the first years of UCR clinical trials services, which was the initiation and performance of the very first registry-based randomized clinical trials (R-RCT). These first trials were based on cluster randomization of hospitals to registry-based quality development or usual care and nicely showed the superior results with registry-based care (20, 21). During the last few years the concept of R-RCT has been the largest international breakthrough of UCR services which is further discussed in other papers (22, 23).

### Quality registries

Since its start in 2002, the first national competence center for national quality registries—the Swedish Cardiovascular Registries—constitutes a large part of Uppsala Clinical Research Center. The basis for this activity is the development and maintenance of an advanced internet-based technology for registration, reporting, and support for systematic improvement of health care processes. The focus of the work in the registry group is to maintain and further develop the concept of internet-based interactive and intuitive data entry systems, preferably integrated with electronic patient records, and to further improve the online information with interactive as well as imperative illustrative analyses. The purpose is also to promote all centers to be involved in a continuous quality improvement process supported by internet-based tools for ‘evidenced-based health care development’. The specific tasks are accordingly to develop, maintain, and improve the national quality registries within cardiovascular diseases; to expand the analysis and reporting from these registries; and to provide support to other registries (6).

The competence center today has the responsibility for around 20 national quality registries, 10 of which are in the cardiovascular area and around 10 in other areas. Today, the UCR registry activities seem to cover all health care areas in life from before birth (*in-vitro* fertilization registry) to very old age (Senior Alert). The internet-based registries usually include mainly hospital-based clinics in the around 70 hospitals in Sweden. However, the Senior Alert registry accepts data from as many as 40,000 different care units all over the country. The UCR quality registry group started with as few as three software developers but rapidly grew to 10 system developers in 2006 and further increased to around 40 people in 2018. In addition, the group also contains several data managers and statisticians for the integrated statistical and scientific analysis.

The registry group collaborates closely with a large number of physicians and research nurses, project leaders, and registry coordinators over the whole country and even internationally. The group provides the following services: national and international internet-based databases and registries; electronic computer forms and remote online data entry; imports from and transfer to external data sources; interactive, simple, and advanced statistical analysis; online reports with tabular and graphical presentations; education, support, and monitoring; data security; maintenance and support; quality development and projects supported by consultant and internet-based education; and finally internet portals for information, communication, collaboration, and education. UCR’s IT-based registration technology is also used in international projects and in other countries for evaluation of the effects of new treatments in health care. Therefore, UCR is today established as the leading European center for registry-based quality development and evaluation of new medical treatments in cardiovascular care. In addition, the registry technology has over the last years been developed to integrate tools for registry-based randomized clinical trials (R-RCT). The international interest for taking part in this development has currently placed UCR as one of the world leaders in technologies for cost-effective pragmatic randomized clinical trials within the usual health care system (6, 24).

### Biostatistics

Biostatistics and epidemiology, supported by data management, are key resources in all medical research. At the beginning, UCR biostatistics group consisted of three statisticians and two data managers. After five years, the group had grown to contain eight biostatisticians, two epidemiologists, and three data managers. Today the group has once more doubled in size and contains several statisticians at the PhD level and specialists in bioinformatics, genetics, and big data analysis. Currently, the group is one of the largest and most experienced in Sweden for management and analysis of quantitative biological data.

The group provides services in planning, recording, management, analysis, and interpretation of data in all areas of clinical research and also develops new statistical models and innovative online tools for interactive statistical analyses. The activities started mainly with services for cardiovascular research but the group now has experience from collaboration in many different areas. The group is now responsible for basic courses of biostatistics in a biomedical program at Uppsala University. Several of the statisticians and epidemiologists are running their own research programs and are acting as tutors for research students. The epidemiology group has recently grown to establish its own organization as an Epidemiological Center within Uppsala University.

### Administration

During the early years, UCR had a very limited single-person administration, handling personnel, finance, localities, IT services, and general services. However, over the years also this group has had to grow in order to handle the increasing demands from an expanding organization, including IT solutions and IT security, quality assurance, budget planning, accounting, contracts, etc. In addition, new services have been added including a communications officer and publication services. Finally, after a few years it became apparent that an internal quality system was a necessity to provide a solid framework for the continuous work with standardized operational procedures (SOPs) and all other quality routines.

Although starting on a small and improvised scale in the hands of a few dedicated people, all main activities at UCR became over the years well-established and were shown to work with high quality, able to cope with the increasing service demand. Based on a combination of assignments from research groups, research foundations, hospitals, county councils, National Board of Health and Welfare, and pharmaceutical, device, and diagnostic companies, the UCR finances have gradually been growing and during most years kept in a good balance.

### Organization

UCR was thus founded as, and still is, a collaborative project between the Faculty of Medicine at Uppsala University and Uppsala University Hospital within Uppsala County Council. From 2007, UCR has been organized as an independent unit reporting directly to the dean of the Faculty of Medicine and the director of Uppsala University Hospital. The dean of the Faculty of Medicine and the director of Uppsala University Hospital appoint the director of UCR for three years. The board of UCR consists of five voting members (three from Uppsala University, two from Uppsala County Council/Uppsala University Hospital) and the UCR director. A scientific advisory committee has for many years supported the UCR director concerning the strategic planning. The UCR director is further supported by an internal steering group, which has regular meetings concerning planning and performance of the daily operations.

The number of employees at UCR has gradually increased in association with an increasing demand for services. UCR started its activities in 2001 with only around 10–15 employees, in 2006 reaching 40–45 full- or part-time employees, and in 2012 around 100. The UCR finances are managed as a combination of department-based finances within the Uppsala University administration and project-based finances within the Uppsala University Hospital administration. The personnel can thereby be employed either by the University or by the University Hospital. The director of UCR is responsible for the finances of all projects within both administrations. All research groups collaborating with UCR have full responsibility for their own finances. Before the start of collaborative projects, the sharing of costs between the respective group and UCR is established in separate contracts. This organization is ideally suited for its purpose to perform cost-effective clinical research and development by integrating the available resources both in the laboratory-based medical research in the University and in the clinical medicine departments in the hospital environment, with direct access to patients, patient records, registries, and hospital-based resources for clinical investigations and care.

### Future

After almost 20 years of development, UCR has now established a stable infrastructure for support of continuous quality development, clinical trials, and translational research in all areas of health care. Currently, UCR is world-leading in the areas of internet-based interactive registry-supported quality development and registry-based randomized clinical trials (R-RCT). UCR has recently been assigned the task to try to implement the SWEDEHEART registry and clinical trials platform in as many European countries as possible. In addition, UCR is one of the world-leading centers for all types of services in conventional randomized clinical trials. In this area, UCR has pioneered international collaboration on biobanking and omics research in order to make personalized and precision medicine an integral part of global collaborative trials. At present, UCR is therefore well positioned to play a gradually expanding role in the national and international development and improvement of health care.
